# Therapeutic Uses and Pharmacological Properties of Garlic, Shallot, and Their Biologically Active Compounds

**Published:** 2013-10

**Authors:** Peyman Mikaili, Surush Maadirad, Milad Moloudizargari, Shahin Aghajanshakeri, Shadi Sarahroodi

**Affiliations:** 1 Department of Pharmacology, Faculty of Pharmacy, Urmia University of Medical Sciences, Urmia, Iran; 2 Urmia University, Faculty of Veterinary Medicine, Urmia, Iran; 3 Department of Physiology and Pharmacology, School of Medicine, Qom University of Medical Sciences, Qom, Iran

**Keywords:** *Allium hirtifolium*, *Allium sativum*, Garlic, Pharmacological effects, Shallot, Traditional uses

## Abstract

***Objective(s):*** Garlic (*Allium sativum* L. family Liliaceae) is well known in Iran and its leaves, flowers, and cloves have been used in traditional medicine for a long time. Research in recent decades has shown widespread pharmacological effects of *A. sativum* and its organosulfur compounds especially Allicin. Studies carried out on the chemical composition of the plant show that the most important constituents of this plant are organosulfur compounds such as allicin, diallyl disulphide, S-allylcysteine, and diallyl trisulfide. Allicin represents one of the most studied among these naturally occurring compounds. In addition to *A. sativum*, these compounds are also present in *A. hirtifolium* (shallot) and have been used to treat various diseases. This article reviews the pharmacological effects and traditional uses of *A. sativum*, *A. hirtifolium*, and their active constituents to show whether or not they can be further used as potential natural sources for the development of novel drugs.

***Materials and Methods: ***For this purpose, the authors went through a vast number of sources and articles and all needed data was gathered. The findings were reviewed and classified on the basis of relevance to the topic and a summary of all effects were reported as tables.

***Conclusion:*** Garlic and shallots are safe and rich sources of biologically active compounds with low toxicity. Further studies are needed to confirm the safety and quality of the plants to be used by clinicians as therapeutic agents.

## Introduction

**Figure 1:**
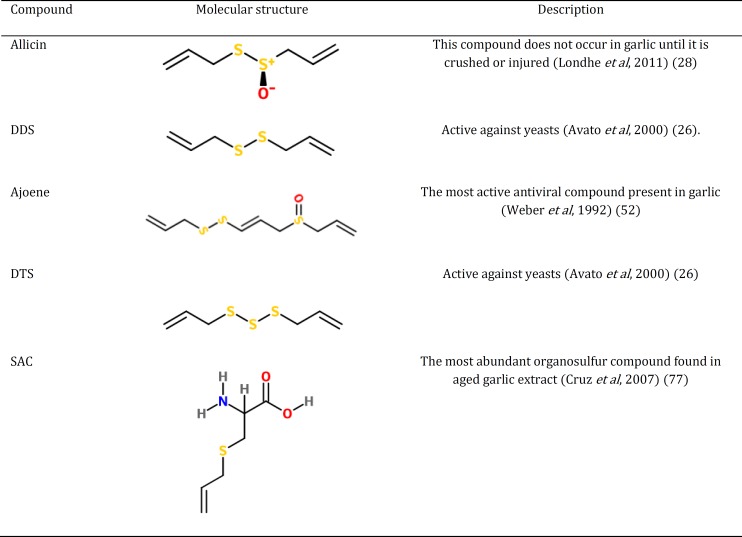
Molecular structure of important organosulfur compounds from *Allium* *sativum* and *Allium hirtifolium*

Garlic (*Allium sativum* L. family Liliaceae) is originally from Asia but it is also cultivated in China, North Africa (Egypt), Europe and Mexico. It is well known in Iran and various parts of this plant have long been used in traditional folk medicines of Iran and some other cultures. It is also used as a spice and food additive (1, 2). The plant is a bulb growing to 25-70 cm with hermaphrodite flowers (3). Leaves and cloves of *A. sativum *have been used in traditional medicine of Iran and other countries for a long time (4, 5). In pharmacological research, there is a lot of evidence about a wide spectrum of pharmacological effects of *A. sativum* and its active compounds with low toxicity (Table 1) (1). Studies carried out on the chemical composition of the plant show that sulfur compounds such as Allicin are important constituents of the plant (6). Although allicin (diallyl-dithiosulfinate) is the most important alkaloid that is generally claimed to be responsible for their beneficial effects and numerous studies have been conducted so far (7), it is pointed out that other sulfur compounds such as diallyl disulphide (DDS), S-allylcysteine (SAC) and diallyl trisulfide (DTS) also have some roles in the effects of the plant (Figure 1) (8). In addition to *A. sativum*, allicin, ajoene and other organosulfides are present in *A. hirtifolium* and play important pharmacological roles (Table 2) (9). This article reviews the pharmacological effects and traditional uses of *A. sativum*, *A. hirtifolium*, and their active constituents.

## Materials and Methods

***Data sources and data extraction***

In order to gather the needed information, systematic literature searches were conducted on MEDLINE, EMBASE, and BIOSIS databases. A vast number of papers (more than 200 original articles and reviews) were studied during the years 2011-2013 and data extraction was performed methodologically based on previously identified keywords including: *Allium sativum*, garlic, *Allium hirtifolium*, shallot, organosulfur compounds, allicin, and ajoene. The dates of articles used as references ranged from 1973 to 2012. Discrepancies were settled through discussion for a final period of 3 months.

***Data presentation***

 The findings were interpreted and classified on the basis of relevance to the topic and a summary of all effects were reported as tables. Each topic starts with a brief review of the traditional uses of the plant that suits the topic (if present) and then the information is supported by the results of various pharmacological studies conducted in that field. The molecular structures of every active compound present in the studied plants were drawn using the online service (www.emolecules.com). Finally based on the reviewed information a conclusion was reached.

***Anti microbial effects***

*Antibacterial*

 Allicin and other sulfur compounds are thought to be the major compounds responsible for the antimicrobial effect of garlic. Garlic is effective against a number of gram-negative, gram-positive, and acid-fast bacteria, including *Staphylococcus*, *Salmonella*, *Vibrio*, *Mycobacteria*, and *Proteus* species (7). Aqueous, ethanol and chloroform extracts of garlic inhibited the growth of the pathogenic bacteria, though with varying degrees of susceptibility. The gram positive *Staphylococcus aureus *was more susceptible to the toxic effects of garlic than its gram negative counterparts. It has been shown that the aqueous extract of garlic can be used alongside conventional antibiotics to fight agents of nosocomial infections that are so prevalent in hospitals (5). An *in vitro* study on the effects of aqueous and ethanolic extracts of garlic against specific bacteria such as *Escherichia coli *and *sal. typhi* showed that the aqueous extract had little or no inhibition while the ethanolic extract had a higher inhibitory effect. Allicin in its pure form was found to exhibit antibacterial activity against multidrug-resistant enterotoxicogenic strains of *E. coli* (10). In another study, the aqueous extract exhibited antibacterial activity against gram positive (*Bacillus subtilis*, *Staph. aureus*) and Gram negative (*E. coli* and *Klebsiella pneumoniae*) strains, while methanol extract showed antimicrobial activity against all the tested microorganisms except *Stap. aureus* (11).

 Garlic ethanolic extract showed maximum activity against *B. subtilis *(12). Allitridi, a proprietary garlic derivative, has been successfully used to treat systemic bacterial infections (such as *Helicobacter pylori*) in China (13). It was shown in another study that the extract of garlic strongly inhibits *Sal. enteritidis*; however *Staph. aureus* showed less sensitivity (14). It has been shown that Gram-negative diarrheagenic pathogens (*E. coli, Shigella sp, Salmonella sp*, and *Pro. mirabilis*) from stool samples were highly sensitive to garlic (2). The significant antibacterial activity of garlic extract on streptomycin-resistant strains (Gram-positive *Staph. aureus* and Gram-negative *E. coli*) solely and in synergism with streptomycin has also been proved (15). In a study by Lai and Roy, fresh extracts of *A. sativum* (garlic) and *Nigella sativum* (black cumin) had more antibacterial activity against the isolates of the urinary tract infection, compared to the individual extract or drugs, such as cefalexin, cotrimoxazole, and nalidixic acid (16). Garlic has antibacterial activity against the pig pathogen *Actinobacillus pleuropneumoniae* serotype 9. The main compound that is suggested to be responsible for this effect of garlic is volatile allyl methyl sulfide (AMS) as a lead compound of volatile garlic metabolites (17). Garlic extract was also effective against *Streptococcus mutans* when tested both *in vitro* and *in vivo*. As *Strep. mutans* is one of the primary aetiological organisms in dental caries development (18), garlic extract mouth rinse might be used effectively in the prevention of dental caries (19).

*Antifungal*

 Allicin (diallyl-dithiosulfinate), which is produced by the garlic enzyme alliinase from the alliin, has been shown to have wide-range antifungal specificity. An *in vivo* study showed that antibody-alliinase conjugates and alliin are effective against murine pulmonary aspergillosis (20). One study showed that allicin from garlic has antifungal activity particularly against *Candida albicans* (10). Another *in vitro* study showed both intrinsic antifungal activity of allicin and its synergy with the azoles, in the treatment of candidiasis (21). Studies on the effect of Amphotericin B (AmB) against *C. albicans *showed that allicin enhances significantly the effect of AmB against *Candida albicans*, *Saccharomyces cerevisiae* and against *Aspergillus fumigatus in vitro* and *in vivo* (22, 23). It was found in another study that polymyxin B (PMB), is effective against various yeasts and filamentous fungi when used in combination with allicin. This combination increases the plasma membrane permeability in *Saccharo cerevisiae*. Swollen spherical structure of the yeast disappeared as a result of structural alterations of its vacuole caused by the synergistic activity between PMB and allicin combination (24). A study showed the effects of diallyldisulphide (DADS), one of the components of garlic, on antioxidant systems in *Candida* species. Changes in antioxidant metabolites and antioxidant activity in the presence of DADS were found in *C. albicans* and *C. tropicalis*. DADS caused a decrease in the activity of all antioxidant enzymes except catalase (25). One study showed that six different mixtures of garlic distilled oils containing diallyl disulfide(DDS) and diallyl trisulfide (DTS), are active against a number of yeasts (*C. albicans*, *C. tropicalis* and *Blastoschizomyces capitatus*) (26). Saponins from *A. sativum* were shown to be effective against *Botrytis cinerea* and* Trichoderma harzianum* (8). Essential oil vapors from *A. sativum* also have inhibitory activity against *Ascosphaera apisin in vitro* (27). In one study, allicin was shown to be more potent in the growth inhibition of *C. albicans* and also suppression of HWP1 gene expression in comparison with fluconazole, a commonly used antifungal. This compound does not occur in garlic until it is crushed or injured (21, 28). Ajoene, another constituent of garlic, is responsible for many pharmacological activities of this plant specially its antifungal effect (29). This substance is more effective in association with antifungal drugs (sulfametoxazol/ trimethoprim) in the treatment of mice intratracheally infected with *Paracoccidioides brasiliensis* (30). In an *in vitro* study the growth of both *Asper. niger* and *C. albicans* were inhibited by ajoene at <20, ug/ml (31). High zones of inhibition were noted with ethanol extracts of *A. sativum* tested against dermatophytes, saprophytes, and Candida species isolated from infected hospitalized patients (32). It has been proven that the blockage of lipid synthesis by aqueous extracts of garlic plays an important role in the anticandidal activity of this plant (33). Alcoholic extracts also have potential anticryptococcal activity against murine disseminated cryptococcosis (34). Another study also showed the sensitivity of *Cryptococcus neoformans* against *A. sativum* (35). A novel antifungal protein, designated allivin, was isolated from *A. sativum* with antifungal activity against *Botrytis cinerea*, *Mycosphaerella arachidicola* and *Physalosporapiricola* (36).

*Anti-parasitic*

An ultrastructural study showed that allicin is able to produce morphological changes in the male *Schistosoma mansoni* (37). Another study indicated that Allicin has antiparasitic activity against *Plasmodium falciparum *and *Trypanosoma brucei brucei* (38). It is also effective against some major human intestinal protozoan parasites such as *Entamoeba histolytica* and *Giardia lamblia *(10). Diallyl trisulfide is a chemically stable final transformation product of allicin. The activity of diallyl trisulfide was investigated against several important protozoan parasites *in vitro*. The results indicated that the compound has the potential to be used in treatment of several human and animal parasitic diseases such as *Trypanosoma sp, Ent. histolytica* and *Giar. lamblia *(39). Ajoene isolated from *A. sativum* is an inhibitor of human glutathione reductase and *Trypa. cruzi *trypanothione reductase. The antiparasitic and cytostatic actions of ajoene may at least in part be due to the multiple effects on key enzymes of the antioxidant thiol metabolism (40). Alchinal is a preparation of three different substances including *Echinacea purpurea* and *A. sativum* extracts and cocoa. It has been demonstrated that this preparation significantly decreases the number of adult forms and muscular larvae of *Trichinella spiralis*. It was demonstrated that after Alchinal administration, the number of adult forms and muscular larvae of this parasite was significantly decreased (41). Garlic oil is effective against a wide range of microorganisms including *Plasmodium spp*,* Trypanosoma spp*,* Leishmania spp*,* Giardia spp*, and *Cochlospermum planchonii *(). Its aqueous extract has been shown to be effective against hymenolepiasis and giardiasis also (43). In an *in vitro* study the ethanol, dichloromethane and water extracts of *A. sativum* were shown to have anthelmintic activity against* Haemonchus contortus* from sheep. The ethanol extract was the most effective in decreasing larval count (44). Another study showed that garlic is effective against nematodes. Aqueous extract from garlic has good activity against *Trichuris muris *and *Angiostrongylus cantonensis* when followed by chloroform extract (45). Garlic is an ingredient of a mixture (Prepared from the extracts of coconut, onion, garlic, fig, date tree, chicory, ananas, and cistrose) tested *in vivo* and *in vitro* for its anthelmintic activity against cestodes (*Hymenolepis diminuta*, *H. microstoma*, and*Taenia taeniaeformis*) and trematodes (*Fasciola hepatica*, *Echinostoma caproni*). In all *in vitro* tests, the target parasites died. In addition, the same composition was effective against the intestinal fluke *Echino caproni*, but not against the liver fluke *F. hepatica* in the final host, while both worms were killed *in vitro* (46). Essential oil of *A. sativum* has paralytic effect on *F. gigantica*. The essential oil produced significant reduction in the frequency and the amplitude of the spontaneous muscular activity of whole fluke at 1 and 3 mg/ml concentrations (1). The extract of *A. sativum* also possesses mosquito larvicidal properties. It is effective against filarial mosquito *Culex quinquefasciatus* (after 24 hr treatment) (47), *Cul. quinquefasciatus *and *Anopheles stephensi* (48). Essential oil from *A. sativum* has acaricidal activity against *Rhipicephalus (Boophilus) microplus (Canestrini)* tick larvae (49). The insecticidal activity of *A. sativum* against larvae of *Aedes albopictus (Skuse) *(50), *Lycoriella ingénue* (51), and *Spodoptera litura *(1000 ppm) has also been shown (11).

*Antiviral*

*A. sativum* has been shown to have antiviral activity. In one study the virucidal activity of this plant was attributed to the following contents in this order: ajoene > allicin > allyl methyl > thiosulfinate > methyl allyl thiosulfinate (52). Also Allicin, the main constituent of *A. sativum*, has a variety of antimicrobial activities both *in vitro* and *in vivo*. Among the viruses which are sensitive to garlic extracts are the human* Cytomegalovirus *(HCMV), influenza B virus, *Herpes simplex virus *type 1, *Herpes simplex virus *type 2,* Parainfluenza virus *type 3*, vaccinia virus,* vesicular stomatitis virus*, *and *human Rhinovirus *type 2 (10). One study showed that Allicin-containing supplements can prevent attacks by the common cold virus (53). The main antimicrobial effect of Allicin is due to its chemical reaction with thiol groups of various enzymes, e.g. alcohol dehydrogenase (10). In an *In vivo* study the administration of garlic in mice models protected them against intranasal inoculation with influenza viruses and enhanced the production of neutralizing antibodies when given the vaccine (7).

 Ajoene, isolated from extracts of garlic may inhibit adhesive interaction and fusion of leukocytes (54). In a study investigating the effect of Allitridin (diallyl trisulfide, a compound from *A. sativum* extraction) on the replication of HCMV and the expression of viral immediate-early genes, it was revealed that this substance has anti-HCMV efficacy (55). In another study, it was supposed that the antiviral activity of garlic in humans may be secondary to a direct toxic effect on viruses. It also enhanced NK-cell (Natural killer-cell) activity that destroys virus-infected cells (7).

***Cardiovascular effects***

*Antihypertensive*

 A statistical study showed that individuals whose blood pressures are on the lower side are more likely to consume more garlic in their diets (56). Various epidemiologic studies have indicated an inverse correlation between garlic consumption and progression of cardiovascular disease (57). The authors are of the opinion that garlic is effective in treatment of mean systolic blood pressure but not d-penicillamine (58).

 In one study the aqueous garlic extract (AGE) caused a decrease in blood pressure and bradycardia by direct mechanism not involving the cholinergic pathway, suggesting a likely involvement of peripheral mechanism for hypotension (59). Another study showed that AGE prevents oxidative stress, systolic blood pressure, aortic NAD(P)H oxidase activity and vascular remodeling in rats with metabolic syndrome (60).

 It has been also shown that preparations of garlic may be tentatively used as an adjunct agent in treatment of arterial hypertension because of its hypolipemic and antioxidant properties (61). *In vivo* and *in vitro* ischemia reperfusion studies have shown that prophylactic administration of AGE prior to ischemia reperfusion inhibits lipid peroxidation and prevents depletion in glutathione through its compounds that led to functional recovery. Its ability to inhibit neutrophil migration could suppress fibrosis formation. These preventive effects are seen in studied model organs such as kidney and liver with functional recovery. Organ system specific activity such as angiotensin converting enzyme-inhibiting action contributes to a cardioprotective and blood pressure lowering effect of garlic (62). The authors are of the opinion that the blood pressure lowering effect of garlic in rats (two-kidney one-clip model) may be partly mediated through the nitric oxide (NO) pathway, by enhanced NO synthesis (63).

A study on the effects of two garlic sources has the potential to reduce systolic blood pressure. The effect of aged garlic extract was accompanied by a decrease of pulse pressure (PP), suggesting an improvement of the pliability of the artery, although raw garlic (RG) powder did not affect PP. However, harmful effects were observed in the RG group, including a decrease in erythrocytes, an increase in reticulocytes, and generation of papilloma in the forestomach (64). Another study showed that garlic is a potent vasorelaxant and could reduce the atherogenic properties of cholesterol (65).

A small pilot study indicated the potential ability of aged garlic extract to inhibit the rate of progression of coronary calcification (66). In a study garlic appeared to be a good adaptogen to be utilized in patients with coronary artery disease (67). One study indicated that increased intake of garlic has been associated with reduced mortality in cardiovascular patients or reduced incidence of myocardial infarction, stroke, and hypertension (68). Another study showed that garlic may beneficially affect two risk factors for atherosclerosis--hyperlipidemia and hypertension (69).

One survey suggested that allicin lowered intraocular pressure, in part, by dual actions at the neuroeffector junction (70). Oxidative damage by free radicals has been implicated in the pathogenesis of vascular disease in hypertension. Authors concluded that the total antioxidant status can be significantly improved by treatment with garlic (71). An *in vivo *study indicated that garlic blocks hypoxic pulmonary hypertension and demonstrated a combination of endothelium-dependent and -independent mechanisms for the effect in pulmonary arterial rings (72).

An *in vitro *study showed that intravenous administration of garlic extracts produced dose-dependent and reversible hypotensive and bradycardic effects (73).

In One survey the authors are of the opinion that although H_2_S (hydrogen sulfide) role in blood pressure regulation and interaction with NO is controversial, H_2_S, through its anti-apoptotic, anti-inflammatory and antioxidant effects, has demonstrated significant cardioprotection. As a result, a number of sulfide-donor drugs, including garlic-derived polysulfides such as diallyl disulfide, diallyl trisulfide and S-ally cysteine, are currently being designed and investigated for the treatment of cardiovascular conditions such as hypertension (74, 75). Stimulation of nitric oxide generation in endothelial cells seems to be the critical preventive mechanism. Cardioprotective effects of dietary garlic are mediated in large part via the generation of H_2_S. Garlic-derived organic polysulfides are converted by erythrocytes into hydrogen sulfide which relaxes vascular smooth muscle, induces vasodilation of blood vessels, and significantly reduces blood pressure (76).

Progressive renal damage and hypertension are associated with oxidative and nitrosative stress. On the other hand, S-allylcysteine (SAC), the most abundant organosulfur compound in aged garlic (AG) extract, has antioxidant properties. The effects of SAC and AG on blood pressure, renal damage, and oxidative and nitrosative stress were studied. The data suggested that the antihypertensive and renoprotective effects of SAC and AG are associated with their antioxidant properties and that they may be used to ameliorate hypertension and delay the progression of renal damage (77). Daily treatment with 600 mg of Allicor (garlic powder tablets) has decreasing effects on both systolic and diastolic blood pressures. It has been shown that time-released tablets of Allicor are more effective in the treatment of mild and arterial hypertension than regular garlic additives (78). Allicin within garlic tablets was shown to be the possible responsible substance for the anti-hypertensive effect of the tablets. Other organo-sulfur compounds may also have a role in the hypotensive mechanisms of garlic (6). It was shown in a study that administration of garlic extract decreases systolic and diastolic blood pressure only in hypertensive animals with no such effect in normotensive ones (79). Allyl methyl sulphide (AMS) and diallyl sulphide (DAS), two garlic derivatives, are shown to inhibit migration and angiotensin II-stimulated cell-cycle progression in smooth muscle cells of aorta. As a result, AMS and DAS may serve as effective antioxidant compounds in the arterial structural changes caused by hypertension (80). Hepatopulmonary syndrome is characterized by the presence of portal hypertension and dilated pulmonary capillaries. In a study, garlic powder and iloprost inhalation demonstrated clinical improvements in the pre- and in the post-transplant period (81).

Administration of moderate doses of garlic along with propranolol has been shown to have beneficial effects in animals with hypertension and myocardial damage (82). Another study indicated that garlic in moderate doses with added hydrochlorothiazide(HCTZ) possesses synergistic cardioprotective and antihypertensive properties against fructose- and isoproterenol-induced toxicities, by increasing the lactate dehydrogenase, creatinine phosphokinase, superoxide dismutase and catalase activities in heart homogenate when used concurrently or separately (83). The influence of garlic on pharmacokinetics of HCTZ was studied. The administration of HCTZ in garlic homogenate pretreated rats was found to decrease the QRS duration, RR interval, QT segment, systolic blood pressure, heart rate, serum potassium level, serum LDH and serum CK-MB activities significantly. It was concluded that careful addition of garlic in moderate doses might result in beneficial effect during treatment of hypertension in patients with myocardial stress as garlic causes substantial fall in excretion of potassium when compared to HCTZ alone treatment in rats (84). One study represented that combination of garlic or its bioactive constituent, S-allyl cysteine sulphoxide, and captopril exerted super-additive (synergistic) interaction with respect to fall in blood pressure and ACE inhibition (85). Another study showed that S-allyl-mercapto-captopril (CPSSA), a conjugate of captopril with allicin, was effective in attenuating systolic and diastolic blood pressures as well as significantly reducing glucose levels (86). A comparable study between the effects of allicin and enalapril on blood pressure (BP) showed similar effects, both of which reduce BP (87).

*Antiatherosclerotic*

One study by Wang and Ng (1999) showed that garlic compounds possess anti-atherosclerotic activity (88). Also numerous animal studies have reported that garlic can have protective effect against atherosclerosis (89). Sulfur-containing volatiles from garlic are the principal compounds responsible for such property and the most abundant volatile compound is diallyl disulfide followed by diallyl trisulfide (90). These active constituent(s) of garlic responsible for its anti-atherogenic action are shown to be mostly present in the oily fraction of the plant (91). Among these constituents, allicin is another compound that plays an important role in anti-atherosclerotic activity of garlic. It is produced upon crushing of the garlic clove. A pure allicin preparation may affect atherosclerosis not only by acting as an antioxidant, but also by other mechanisms, such as lipoprotein modification and inhibition of LDL uptake and degradation by macrophages (92). In a study, 112 patients (47 men and 65 women) 40 to 60 years of age were examined. 56 patients had ischemic heart disease and/or equal disorders. Another 56 patients were free of any signs of atherosclerosis, but had one or more cardiovascular pathology risk factor. Six month therapy using allicor results in moderate hypolipidemic and antioxidative effect. A dosage of 600 mg per day decreases ten-year chance of fatal cardiovascular complications in patients with clinical signs of atherosclerosis, whereas in patients who have no signs of atherosclerosis the complications are decreased with dosage of 300 mg per day (93). Another survey indicated that garlic indirectly affects atherosclerosis by reduction of hyperlipidemia. Moreover, in animal models, garlic causes direct antiatherogenic (preventive) and anti-atherosclerotic (causing regression) effects at the level of artery wall. It was suggested in one study that garlic powder also manifests direct anti-atherogenic-related action not only *in vitro *but also *in vivo* (94). Garlic's direct effect on atherosclerosis may be explained by its capacity to reduce lipid content in arterial cells and to prevent intracellular lipid accumulation. This effect, in turn, is accompanied by other atherosclerotic manifestations, i.e., stimulation of cell proliferation and extracellular matrix synthesis (95). A study demonstrated that garlic reduces the atherogenic properties of cholesterol (65). As sited above, suppressed LDL oxidation may be one of the powerful mechanisms accounting for the anti-atherosclerotic properties of garlic (96, 97). In one study, intake of high-dose garlic powder dragees significantly reduced the increase in arteriosclerotic plaque volume by 5-18% or even caused a slight regression within the observational period of 48 months (98).

 Fish oil and garlic combinations can serve as good dietary supplements with anti-atherosclerotic properties (99). Other possible mechanisms for lipid lowering and anti-atherogenic effects of garlic include inhibition of the hepatic activities of lipogenic and cholesterogenic enzymes that are thought to be the origin for dyslipidemias, increased excretion of cholesterol and suppression of LDL-oxidation (100). In an *in vitro *study, the potential anti-atherosclerotic property of moderate and high doses of garlic homogenate (GH was significantly attenuated by propranolol and hydrochlorothiazide. However, GH anti-hyperlipidemic activity was augmented by captopril (101). Another study indicated that (egg yolk-enriched garlic powder) EGP inhibits copper-induced LDL oxidation in a dose-dependent manner that might be ascribed, in part, to the biodistribution of garlic compounds and egg yolk interaction. This finding suggests that EGP might be useful in the prevention of atherosclerosis (102).

*Antithrombotic*

Garlic extracts and several garlic constituents demonstrated significant antithrombotic actions both *in vitro* and *in vivo*. Allicin and adenosine are the most potent antiplatelet constituents of garlic (103). A study suggested that odorless garlic not only activates fibrinolytic action by accelerating (tissue-type plasminogen activator) t-PA-mediated plasminogen activation, but also suppresses the coagulation system by down regulating thrombin formation, suggesting a beneficial role in preventing pathological thrombus formation in such cardiovascular disorders (104). A study mentioned that aqueous extract of garlic inhibits platelet aggregation induced by several aggregation agents, including arachidonate in a dose-dependent manner (105).

Another survey indicated that garlic extracts act through inhibition of the ADP (adenosine diphosphate) pathway. Their mechanisms of action are comparable to that of the clinically used drug clopidogrel. The pharmacologically active component of the extracts appears to be lipophilic rather than hydrophilic (106). One study mentioned that the aromatic thiosulfonate derived from garlic is a very effective inhibitor of platelet aggregation (107). Diallyl trisulfide (DATS) is one of the major constituents in garlic oil and has demonstrated various pharmacological activities, such as antithrombotic (108). DAT-rich garlic oil showed anticoagulant action due to inhibition and/or inactivation of thrombin, in an animal study. In addition DAT-rich garlic oil benefits blood anticoagulation factors, which might further prevent the development of thrombus formation. However, the intake of garlic oil at high dose significantly increased plasma fibrinogen concentration (*P*<0.05) and affected the levels of several hematological parameters such as erythrocyte count, hemoglobin and platelets (*P*<0.05). Supplementation of garlic oil at 5 mg/kg BW had anticoagulation effect in this study (109). It was shown in a survey that diallyl disulphide (DADS) and DATS - are usual constituents of garlic oil, with antiplatelet activity. They also inhibit platelet thromboxane formation. In this respect DATS is more potent than DADS (110). The antiplatelet activity of methyl allyltrisulfide (MATS), a component commonly present in steam-distilled garlic oil, has also been demonstrated. MATS inhibits arachidonic acid cascade at the reaction site with PGH synthase (111). In a study allicin and thiosulfinates were considered as responsible compounds for the (*in-vitro *antiaggregatory activity) IVAA response. It was also shown that the loss of activity, and the partial loss of antithrombotic effect in crushed-cooked garlic may be compensated by increasing the amount consumed (112). Authors mentioned that sulfur compounds’ contribution to the health promotion in allium species are produced via enzymic and thermal reactions. Potent antithrombotic agents which have been identified as allyl trisulfides, dithiins, and ajoene in garlic are thermochemically transformed forms of allicin (allyl 2-propenethiosulfinate) (113). A study showed that allicin had the strongest antiplatelet activity at 0.4 mM inhibiting aggregation by 89% (114). Ajoene is another potent antiplatelet compound isolated from alcoholic extracts of garlic. It is suggested that ajoene may be potentially useful for the acute prevention of thrombus formation induced by severe vascular damage, mainly in arterial sites with low local shear rates (115, 116). One study indicated that the antiaggregatory effect of ajoene is causally related to its direct interaction with the putative fibrinogen receptors (117). Another survey demonstrated that the antithrombotic potential of ajoene is substantially increased in the presence of physiologically and pharmacologically active antiplatelet agents (118). In a study, ajoene inhibited platelet aggregation induced by arachidonic acid, adrenaline collagen, adenosine diphosphate and calcium ionophore. The nature of the inhibition was irreversible (119). It has been suggested that supplements of garlic could adversely affect coagulation when taken alone or in combination with antiplatelet medications (120). In a study coadministration of aged garlic extract and cilostazol did not enhance the antiplatelet activity compared with individual drugs (121). Another study suggested that aged garlic extract is relatively safe and poses no serious hemorrhagic risk for closely monitored patients on warfarin oral anticoagulation therapy (122).

Spolarich and Andrews mentioned that patients undergoing routine dental and dental hygiene procedures do not need to discontinue the use of anticoagulant and antiplatelet medications (such as aspirin). However, alterations in drug use may be required for those patients undergoing invasive surgical procedures. It is recommended that herbal supplements, such as garlic, must be discontinued 2 weeks prior to receiving invasive surgical procedures (123).

*Blood factors*

One survey mentioned that garlic has antihyperlipidemic, hypocholesterolaemic and hypo triacylglyceride activities (124). The hypoglycemic and hypolipidaemic effects of garlic have been shown in sucrose fed rabbits also (125). In one study, raw and boiled garlic improved plasma lipid metabolism and plasma antioxidant activity in rats. Thus, dietary garlic was effective in reducing the oxidant stress, which was indicated by an increase of antioxidant activity and a decrease of lipids in the rats' blood (126).

In another study, garlic powder significantly (*P* < 0.05) lowered the animal`s blood lipid levels (127). Garlic has been shown to have applications as a hypoglycemic agent (128). A study suggested a new mechanism for the hypolipidemic effect of fresh garlic. Long-term dietary supplementation of fresh garlic may exert a lipid-lowering effect partly through reducing intestinal MTP (microsomal triglyceride transfer protein) gene expression, thus suppressing the assembly and secretion of chylomicrons from intestine to the blood circulation (129). Short-term garlic therapy in adults with mild to moderate hypercholesterolemia does not affect lipid levels (130). In a study the water soluble protein fraction of garlic was investigated for its effect on hyperlipidemia induced by alcohol (3.76 g/kg body wt/day). It showed hypolipidemic action mainly due to an increase in cholesterol degradation to bile acids and neutral sterols and mobilization of triacyl glycerols in treated rats. Garlic protein (500 mg/kg body wt/day) showed significant hypolipidemic action comparable with a standard dose of gugu-lipid (50 mg/kg body wt/day) (131). One study in 1984 showed that garlic oil has hypolipidemic effects in ethanol-fed rats (132). In another study, the water soluble proteins and the essential oil of garlic were investigated for their hypolipidemic effect on hyperlipidemia induced by cholesterol containing diet in albino rats. Both garlic protein (16% of diet) and garlic oil (100 mg/kg body weight/day) exhibited significant lipid lowering effects (133). A survey mentioned that garlic methanol-extracts behave as hypolipidemic drugs, increasing the activity of peroxisomal fatty acyl-coenzyme A oxidase and of total carnitine acetyl-coenzyme A transferase in primary cultures of rat hepatocytes (134). In an *in vivo* study, garlic demonstrated a reduction of lipid plaques in the arteries of hypercholesterolemic animals. It decreased accumulation of cholesterol in vascular walls, and had other positive interventions (135).

In one study, the glutathione reductase activity that was lowered in hypercholesterolemic conditions, methemoglobin concentration that was significantly increased in hypercholesterolemic rats and significant fall in hepatic total thiols in hypercholesterolemia were partially corrected by garlic. Similarly, the lowered activities of hepatic antioxidant enzymes in hypercholesterolemic rats were effectively countered by this plant (136).

Garlic treatment significantly diminished total-cholesterol, LDL-cholesterol and triglycerides, but not HDL-cholesterol in chronic nephrotic syndrome (NS). These data indicate that garlic treatment ameliorates hyperlipidemia and renal damage in chronic NS which is unrelated to proteinuria or antioxidant enzymes (137). In a survey, hepatic triglyceride content that was significantly higher in high-fat fed rats was effectively countered by inclusion of the hypolipidemic spice agents such as garlic in the diet (138).

One study mentioned that garlic's organosulfur compounds (such as diallyl trisulfide) display hypolipidemic effects by inhibiting fatty acid and cholesterol synthesis (139).

Diallyl disulfide, an active principle of garlic (*A. sativum*), is known for its antihyperlipidemic properties (140). Water-soluble organosulfur compounds, S-allyl cysteine (SAC), S-propyl cysteine (SPC) and S-ethyl cysteine (SEC), were studied. The results indicated that SAC, SEC, and SPC inhibit lipid biosynthesis in cultured rat hepatocytes, and further suggested that these S-alk(en)yl cysteines of garlic impair triglyceride synthesis in part due to decreased *de novo* fatty acid synthesis resulting from inhibition of fatty acids (141). Allicin (diallyl disulphide-oxide) exerts various beneficial biological effects such as antihyperlipidemic (142) and hypoglycaemic actions (143). Dietary garlic also reduces the cholesterol gallstone incidence by 15-39 % (144). Action of long-acting garlic powder tablets (Allicor) have been investigated on blood factors. The results show that allicor lowers total cholesterol, LDLP cholesterol, raises HDLP cholesterol and therefore can be recommended for correction of lipid content in patients with moderate hyperlipidemia (145). A comparative study on the beneficial effects of garlic amla (Emblica Officinalis Gaertn) and onion (*A. cepa L*) on hyperlipidemia showed that the order of the curative effects of the vegetables is as follows: garlic > amla > onion (146).

***Anticancer effects***

A study mentioned that phytoalexins have been identified in at least 75 plants including garlic. Preclinical evidence has suggested that these compounds possess anticancer properties including an inhibition of cell proliferation, invasion and metastasis, hormonal stimulation, and stimulatory effects on expression of metabolizing enzymes (147). Diallyl sulfide (DAS), diallyl disulfide (DADS) and diallyl trisulfide (DATS) derived from garlic have been shown to exhibit anticancer activities (148). The cytotoxicity caused by DATS is mediated by generation of ROS (reactive oxygen species) and subsequent activation of the ROS-dependent caspase pathway in U937 leukemia cells (108). DATS has been shown to induce apoptosis in many human cancer cell lines *in vitro* and also affords significant protection against cancer in animal tumor models *in vivo *i.e. colorectal cancer (149). Another suggested that DADS treatment may inhibit tumor cell motility and invasion and therefore, act as a dietary source to decrease the risk of cancer metastasis (150).

Recently, S-allylcysteine (SAC) has been identified as a potent compound derived from garlic. This substance has *in vitro* chemo-preventive activity. It may also be a promising candidate for prostate cancer treatment (151). Allicin (diallyl thiosulfinate), the best-known biologically active component in freshly crushed garlic extract, is effective on cell proliferation of colon cancer cells (152). A study indicated that the anticancer action of aged black garlic extract may be partly due to its antioxidant and immunomodulative effects (153).

***Anti-inflammatory effect***

Garlic extracts have been shown to exert anti-inflammatory effects (154). In one study, garlic treatment significantly attenuated inflammation and injury of the liver induced by *Eimeria papillata* infections (155). The anti-inflammatory activity exhibited by garlic oil is mainly through inhibiting the assembly-disassembly processes of the cytoskeleton (156).

Other authors have shown the preventive effect and possible toxicity of garlic oil and its organosulfur compounds in endotoxin-induced systemic inflammation and intestinal damage (157). A lead compound derived from allicin is shown to be a good starting point for the development of anti-inflammatory drugs with fewer side effects (158).

One study indicated that thiacremonone, a sulfur compound isolated from garlic, inhibits neuroinflammation and amyloidogenesis through inhibition of NF-κB activity, and thus could be applied for intervention in inflammation-related neurodegenerative diseases including Alzheimer's disease (159).

***Immunomodulatory effect***

Immunomodulation is among innumerable biological activities of *A. sativum*. Aged garlic extract has been shown to have superior immunomodula-tory properties over raw garlic extract (160). This effect of garlic is attributed to the transformed organosulfur compounds (161). Aged garlic fructans have recently been shown to possess immunomodulatory activities *in vitro *(160). Garlic extract is concentration-dependently effective on the proliferation of interleukin (IL)-2 and interferon (INF)-γ gene expression of stimulated lymphocytes (162). Garlic extracts reduced macrophage infection through induction of nitric oxide (NO) production *In vitro* (163).

A study demonstrated that immune-mediated liver damage in mice can be prevented by allicin, probably because of its immunomodulatory effects on T cells and adhesion molecules and inhibition of NF-kappaB activation (164). Another observation indicated that allicin exerts an inhibitory immunomodulatory effect on intestinal epithelial cells and it may have the potential to attenuate intestinal inflammation (165). Allicin exerted an *in vitro* immunomodulatory effect on certain functions of the peripheral blood cells (166).

***Toxicology***

Tattelman mentioned that garlic appears to have no effect on drug metabolism, but patients taking anticoagulants should be cautious. It seems prudent to stop taking high dosages of garlic seven to 10 days before surgery because garlic can prolong bleeding time (167).

One study indicated that garlic application usually results in local inflammation, but, if applied under a pressure bandage, or if there is poor wound care or a secondary infection, it can cause a severe dermal reaction and a deep chemical burn (168). Data of a study showed that a high garlic dose induced liver toxicity and a pro-oxidative status characterized by increased malondialdehyde and decreased antioxidant enzyme activities as catalase, peroxidase, and superoxide dismutase (169). Another study suggested that garlic with high dose has the potential ability to induce liver damage (170). 

A parallel study also highlighted the potential ability of a high dose of garlic to induce morphological changes in the liver and kidneys (171). Administration of high doses of garlic (500 mg/kg) results in profound changes in lung and liver tissues of rats. Intraperitoneal administration of the high dose of garlic is more damaging to lung and liver tissue of rats than oral administration (172). It is also shown that the adverse effect of high doses of garlic oil might further influence the hemostatic balance (109).

High doses of diallyl disulfide may further complicate the metabolic disturbances in diabetes (173). High dose of garlic oil worsened intestinal mucosal damage accompanied by elevated peripheral proinflammatory cytokines in another study (157).

***Active compounds***

It has been shown that sulfur compounds such as allicin are important constituents of garlic (6). Although allicin (diallyl-dithiosulfinate) is the most important alkaloid that is generally claimed to be responsible for most of the beneficial effects of the plant (7); however, it is pointed out that other sulfur compounds such as diallyl disulphide (DDS), S-allylcysteine (SAC) and diallyl trisulfide (DTS) also have some roles in the pharmacological effects of the plant (88). SAC is the most abundant organosulfur compound found in aged garlic extract (77). It has also been shown that allicin (diallyl-dithiosulfinate) does not occur in garlic until it is crushed or injured (28). Among the active compounds present in the plant, DTS and DDS are the most active against yeasts (26) and ajoene is the main compound responsible for the antiviral activity of garlic (52).

***Drug interaction and pharmacokinetics***

One study indicated that those who use traditional/complementary/alternate medicines (TCAMs) in addition to antiretroviral (ARV) treatment may be at risk of experiencing clinically significant pharmacokinetic (PK) interactions, particularly between the TCAMs and the protease inhibitors (PIs) and non-nucleoside reverse transcriptase inhibitors (NNRTIs). Mechanisms of PK interactions include alterations to the normal functioning of drug efflux transporters, such as P-gp and/or CYP isoenzymes, such a CYP3A4 that mediate the absorption and elimination of drugs in the small intestine and liver. Specific mechanisms include inhibition and activation of these proteins and induction via the pregnane X receptor. Garlic exhibited potentially significant interactions, each with a PI or NNRTI (174). *In vivo* absorption changes are possible between aged garlic extract and cardiovascular, antidiabetic and antiviral drugs, but the magnitude of the changes depends on the most profound process involved (influx, efflux, passive diffusion) in compound’s permeability (175). In a study, pharmacokinetic interaction of garlic and atorvastatin in dyslipidemic rats was shown (176). Another study indicated that the bioavailability and half-life of propranolol was significantly enhanced by 2- and 3-folds, respectively, in animals pretreated with garlic (250 mg/ kg) (82). It has been also shown that herbs such as garlic with the potential to significantly modulate the activity of drug-metabolizing enzymes (notably cytochrome p450 isozymes) and/or the drug transporter P-glycoprotein participate in potential pharmacokinetic interactions with anticancer drugs (177).

**Table 1:** Pharmacological effects of *Allium sativum*

System	Effect	Preparation	Reference
Antibacterial	*Staphylococcus aureus*	Aqueous, ethanol, chloroform extract	EL-mahmood, 2009 (5)
	*Escherichia coli, Salmonella typhi*	Aqueous and ethanolic extract	Ankri and Mirelman, 1999 (10)
	*Bacillus subtilis, Kelebsiella pneumoniae*	Aqueous, methanol and ethanol extract	Meriga *et al*, 2012; Pundir *et al*, 2010 (11, 12)
	*Helicobacter pylori*	Extract	Liu *et al*, 2010 (13)
	*Sal enteritidis*	Extract	Benkeblia, 2004 (14)
	*Shigella sp, Proteus mirabilis*	Extract	Eja *et al, *2007 (2)
	*Actinobacillus pleuro pneumonia serotype 9*	Extract	Becker *et al*, 2012 (17)
	*Streptococcus mutan*	Extract	Loesche, 1986 (18)
Antiviral	*Human cytomegalo virus(HCMV),* *Influenza B, Herpes simplex virus type 1-2, Parainfluenza virus type 3, vaccine virus, Vesicular stomatitis virus, Human rhino virus type *2	Not mentioned	Ankri and Mirelman, 1999 (10)
Antifungal	*Candidia albicans, C. tropicalis, Blastoschizomyces capitatus*	Extract	Avato *et al*., 2000 (26)
	*Botr. cinerea, Trichoderma harzianum*	Extract	Lanzotti *et al*., 2012 (8)
	*Ascosphaera apis*	Essential oil vapors	Kloucek *et al*., 2012 (27)
	*Paracoccidioides brasiliensis*	Extract	Thomaz *et al.*, 2008 (30)
	*Aspergillus niger*	Extract	Matsuura and Nakagawa, 1987 (31)
	*Dermatophytes, saprophytes*	Ethanol extract	Shamim *et al.*, 2004 (32)
	*Cryptococcal* *Botr. cinerea, Mycosphaerella arachidicola, Physalospara piricola*	Alcoholic extract Extract	Khan and Katiyar, 2000 Wang and Ng, 2001 (34)
Anti-parasitic	*Trypanosoma sp, Entamoeba hirtolytica, Giardia lamblia*	Extract	Lun *et al,* 1994 (39)
	*Trypa. Cruzi* *Plasmodium spp, Giardia spp,*	Extract	Gallwitz *et al,* 1999 (40)
	*Leishmania spp, Cochlospermum planchomi*	Extract	Anthony *et al,* 2005 (42)
	*Hymenolepiasis, Giardiasis*	Aqueous extract	Soffar and Mokhtar, 1991 (43)
	*Haemonchus contortus*	Ethanol, dichloro methane and water extract	Ahmed *et al,* 2012 (44)
Cardiovascular	Hypotensive via increasing nitric oxide synthesis	Extract	Al-Qattan *et al,* 2006 (63)
	Hypotensive (endothelial dependent and independent)	Not mentioned	Fallon *et al,* 1998 (72)
	Induces vasodilation with H_2_S	Extract	Ginter and Simko, 2010 (76)
	Angiotensin converting enzyme-inhibiting activity	Aqueous extract	Sener *et al*, 2007 (62)
	Stimulation of nitric oxide generation in endothelial cells	Garlic derived polysulfides	Ginter and Simko, 2010 (76)
	Bradycardia	Aqueous extract	Nwokocha *et al,* 2011(59)
	Hepatopulmonary syndrome	Garlic powder	Theveno *et al,* 2009 (81)
	Decreases systolic blood pressure	Aged garlic	Harauma and Moriguchi, 2006 (64)
	Vasorelaxant	Not mentioned	Zahid Ashraf *et al*, 2005 (65)
	Coronary artery disease	Extract	Verma *et al*, 2005 (67)
	Reduce myocardial infarction, Shoke	Not mentioned	Yang *et al*, 2011 (68)
	Anti-thrombotic	Extract and derived	Choi and Park, 2012 (108)
	Anti-atherosclerotic	Volatiles derived	Calvo-Gómez *et al,* 2004 (90)
Blood	hypolipemic effect	Capsule of garlic preparation	Duda *et al*, 2008 (61)
	Hypolipidemia, Hypocholesterolaemic, Hypotriacylglyceride	Extract	Kuda *et al,* 2004 (124)
	Hypoglycemic	Extract	Sengupta *et al*, 2004 (128)
	Hypolipidemic	Organosulfur compound	Lii *et al*, 2012 (139)
Immune system	Immunomodulation	Extract	Chandrashekar and Venkatesh, 2012 (160)
All	Anti-inflammatory	Extract	Ben *et al,* 2012
	Antioxidant properties	Organo sulfur compound in aged garlic	Cruz *et al,* 2007 (77)

***Allium hirtifolium***

*Antimicrobial*

 It has been shown that the alcoholic and aqueous extracts of shallot (*A. hirtifolium*) have good antifungal activity against *Aspergillus fumigatus, Asper. flavus, Asper. niger, Penicillium gryseogenum, Alternaria, Microsporum canis* and *Trichophyton mentagrophytes* in comparison with the miconazole (178). A comparative study between Persian shallot aqueous extract and chlorhexidine on salivary bacterial counts indicated that shallot extract has more persistent inhibitory action than chlorhexidine mouth rinse lasting up to 24 hr (179). 

In One study *A. hirtifolium *exhibited significant anti-trichomonas activity due to its components such as allicin, ajoene and other organosulfides, comparable to metronidazole (9).

*Anticancer*

One study showed that components of *A. hirtifolium* can dose-dependently inhibit proliferation of tumor cell lines. Therefore, *A. hirtifolium* might be a candidate for tumor suppression (180).

***Clinical trials (garlic)***

By a simple search in the literature it could be figured out that garlic has been used clinically to elicit desirable pharmacological and therapeutic effects. For instance, the use of garlic along with iloprost improved both the pre and post-transplant period in patients with hepatopulmonary syndrome (81). It has also been shown that daily administration of allicin may decrease the occurrence of fatal cardiovascular complications in atherosclerotic patients (93). Although garlic has the potential to be used clinically in the treatment of some disorders, care should be taken regarding its usage with other medications due to possible drug interactions that might arise as a result (174).

**Table 2:** Pharmacological effects of* Allium hirtifolium*

System	Effect	Reference
Antibacterial	Antibacterial: *Staphylococcus spp,* *Salmonella spp,* *Nibrio spp,* *Mycobacteria spp,* *Proteus spp*	Tariq *et al.*, 1988 (7)
	*Escherichia coli*	Ankri and Mirelman, 1999 (10)
Antiviral	*Common cold virus*	Josling, 2001 (53)
Antifungal	*Candida albicans (Candidiasis)*	Ankri and Mirelman, 1999 (10)
	*Saccharomyces cerevisia*	Khodavandi *et al*., 2010 (21)
	*Aspergillus fumigatus*	An *et al*., 2009; Ogita *et al,* 2006 (23)
Antiparasitic	*Schistosoma mansoni* (male) *Plasmodium falciparum,* *Trypanosoma brucei brucei*	Lima *et al*, 2011 (37)
	*Entamoeba histolytica, Giardia lamblia*	Ankri and Mirelman, 1999 (10)
Cardiovascular	Hypotensive intraocular pressure	Chu* et al*, 1993 (70)
	Hypotensive	Younis *et al*, 2010 (86)
	Anti-Atherosclerotic	Gonen *et al*, 2005 (92)
	Antithrombotic or Anti-aggregatory	Cavagnaro *et al*, 2007 (112)
Blood	Hypolipidaemic	Sela *et al*, 2004 (142)
	Hypoglycaemic	Mathew and Augusti, 1973 (143)
Immune system	Immunomodulatory	Bruck *et al*, 2005 (164)
CNS	Neuro protection	Zhu *et al*, 2012 (182)
Respiratory system	Pulmonary oedema	Krumm *et al*, 2012 (183)
Other	Anticancer	Bar-chen *et al*, 2010 (152)
	Anti-inflammatory	Krishna and Yadav, 2012 (158)
	Antidiabetic	Younis *et al*, 2010 (86)

## Discussion

Our aim in preparing this paper was to show the traditional usage and previously confirmed pharmacological effects of garlic along with shallots as two of the most well-known medicinal plants in Iran and to illustrate their potential to be used as novel sources for development of new drugs based on the most recent associated studies. As it is shown in this study, garlic has a wide range of pharmacological effects including antimicrobial, cardiovascular, anti-inflammatory, anticancer, and immunomodulatory activity among many other effects. Organosulfur compounds present in garlic and shallots are the most important contents responsible for most of their pharmacological effects. Among these biologically active compounds, allicin, allyl methyl sulfide, DTS, and ajoene have been shown to be the main responsible compounds for the antifungal, antibacterial, antiprotozoal, and antiviral effects of garlic, respectively. It is evident from this study that *A. sativum* may exert toxicity only at high doses and that there have been few reports of intoxications following the ingestion of garlic. However, care should be taken by scientists and clinicians regarding usage of this plant for therapeutic purposes until adequate studies confirm the safety and quality of the plant. 

## Conclusion

Finally based on this information, this review provides the evidence for other researchers to introduce garlic and shallots and their sole active compounds as safe and effective therapeutic sources in the future.
